# Participation of father in perinatal care: a qualitative study from the perspective of mothers, fathers, caregivers, managers and policymakers in Iran

**DOI:** 10.1186/s12884-018-1928-5

**Published:** 2018-07-11

**Authors:** Vahideh Firouzan, Mahnaz Noroozi, Mojgan Mirghafourvand, Ziba Farajzadegan

**Affiliations:** 1 Student Research Center, Faculty of Nursing and Midwifery, Isfahan University of Medical Sciences, Isfahan, Iran; 2 Department of Midwifery and Reproductive Health, School of Nursing and Midwifery, Isfahan University of Medical Sciences, Isfahan, Iran; 3 Social Determinants of Health Research Center, Tabriz University of Medical Sciences, Tabriz, Iran; 4 Department of Community Medicine, Medicine School , Isfahan University of Medical Sciences, Isfahan, Iran

**Keywords:** Men, Participation, Perinatal care, Iran

## Abstract

**Background:**

The participation of the father during the perinatal period is an important strategy for improving the mother’s health. Very few studies exist about the father’s role in promoting the health of the mother in Iran; thus, the present study was conducted to examine the role of fathers in perinatal care.

**Methods:**

The present study was a qualitative research. Participants were selected using purposeful sampling with maximum variation and the data was collected through in-depth interviews, focus group discussions and field notes. The data was analyzed using conventional content analysis.

**Results:**

After data analysis, the main categories extracted were: “help in maintaining the health of the mother and fetus”, “emotional support of mother”, “comprehensive participation of father in married life”, “preparing for safe delivery” and “postpartum support”.

**Conclusions:**

The results of the present study provide different perspectives on the participation of fathers when designing culture-based intervention for their participation in perinatal care.

**Electronic supplementary material:**

The online version of this article (10.1186/s12884-018-1928-5) contains supplementary material, which is available to authorized users.

## Background

Pregnancy is one of the most important, stressful and yet glorious periods of a woman’s life [1]. The emotional and physical health of women during this period has significant effects on fetal health, successful vaginal delivery and breastfeeding [2]. In the International Conference on Population and Development (ICPD) and the Fourth World Conference on Women emphasized the positive role of fathers in the reproductive health and rights of women [3] and have proposed this as a human health rights priority [4]. The World Health Organization (WHO) reports that, every 2 min, one woman dies from pregnancy- and delivery-related complications [5], but 99% of these deaths could be prevented [6]. WHO has defined the father’s participation in safe motherhood programs as facilitating access to and use of perinatal care, increasing awareness and participating in delivery preparation programs [7]. Women who benefit from the support of their husbands during pregnancy feel more empowered to tolerate the pressure and difficulties of pregnancy, better tolerate labor and more easily adapt to having an infant after delivery [8].

Davis reported that, in Australia, the father’s participation in counties having medium and low incomes was significantly related to the mother’s mortality during delivery [9]. Also, other studies have reported the positive effects of father participation [10–13] as including a decrease in smoking and alcohol consumption, the risk of preterm labor, low birth weight, intrauterine growth restrictions and infant mortality [14, 15].

In recent years, the willingness of fathers to participate in women’s health has increased [16], but policies for recognizing the role of fathers in the health of the mother, infant and child exist in the health system of only a few countries. Studies show that, even in regions where these policies exist, such policies have not been implemented [17]. In Iran, as in other countries, the government has considered common approaches to the care of pregnant women, but no policies or programs exist that encourage participation of fathers during the perinatal period. In recent years, because of efforts to encourage women to have vaginal delivery, eight delivery preparation classes are conducted for pregnant women that include one session in which the fathers participate. Practically, however, this is executed at a limited number of health centers and problems exist in its execution. From the perspectives of women’s health and social welfare, increasing the participation of fathers in the perinatal period is considered a positive matter. However, recognition of the sensitive nature of gender roles in different cultures is necessary when studying the attitudes of the beneficiary individuals and groups about this subject. Because qualitative research is an approach to discovering and describing the experiences of participants and conceptualizing them, it could increase insight and awareness about human experiences. This approach is usually used when there is a need for explaining concepts and their relationships [18]. The present qualitative study was conducted to determine the concept of father participation in perinatal care from the perspectives of the mothers, fathers, caregivers, managers and policymakers.

## Methods

### Study design

The present qualitative research used a content analysis approach.

### Settings, sample and recruitment

The participants were 12 women who were pregnant or had just delivered residing in Tabriz, Iran, six husbands of pregnant women or women who had recently experienced delivery, 19 health care providers (midwives, gynecologists and nurses), three deputy health managers at Tabriz University of Medical Sciences and five policymakers from the Ministry of Health and Medical Education who were selected through purposive sampling. The mothers and fathers were accessed through postpartum wards of hospitals and perinatal clinics and midwife or gynecologist offices. The inclusion criteria were willingness to participate in the study, providing informed consent, having at least 5 years of working experience with caregivers, being able to understand and express their experiences, having Iranian nationality and being able to understand and speak Farsi language. The demographic characteristics of the 45 participants are shown in Table 1.

**Table 1:** Participants’ demographic characteristics

Age	15–55
Gender	female (36), male (9)
Educational level	middle school degree (1), diploma (5)
	associate’s and bachelor’s degree (17)
	master’s degree and Ph.D (22)
Occupational status	employee (35), housewife (8), freelancer (2)
Number of children	0–3
Pregnancy status of the participant or their spouse	delivered (28), pregnant (13)
	with a history of pregnancy or delivery (1)
	without a history of pregnancy or delivery(3)

### Data collection

The data was collected through face-to-face in-depth interviews, focus group discussions and field notes between July and November 2017. Interviews began with the open question of “How can fathers participate in the care of their wives during pregnancy, delivery and after delivery? Please explain.” The course of the interviews was guided by the participants’ open and interpretative answers. In addition, as the interviews went on, more detailed questions (based on the interview guide) were asked (see Additional files 1, 2, 3, 4, 5 and 6 for copies of the topic guides). In the present study, 45 interviews (25 to 100 min) took place at a location of the participant’s choice. Two focus group discussions (one for eight pregnant women and one for seven caregivers) were conducted. The interviews continued until data saturation occurred.

### Data analysis

The data was analyzed using conventional content analysis [19]. The interviews were in Persian and were recorded using an mp4 device. The researcher regularly transliterated the recordings. The text was reviewed repeatedly by the first author (VF) to achieve a comprehensive understanding of the interviews. The sentences and phrases were coded using the inductive method, those with similar codes were merged, those with similar meanings were put into the same categories and sub-categories were created. The sub-categories were compared using the inductive method, and similar sub-categories were put into the same main category.

### Rigor and trustworthiness

To provide credibility to the results, in-depth interviews were carried out at different times and places and a combination of data gathering methods were used when selecting participants. These were personal interviews, focus group discussions, maximum variation of educational level, socioeconomic condition, occupation, age, number of pregnancies and deliveries. To confirm the accuracy of the data, it was shared with four participants at different sessions and their opinions were sought to conduct a member check. The opinions of four experts were also sought to confirm consistency between the collected data and the participant statements. To increase transferability, the results of the study were given to three individuals with characteristics that were similar to the participants, but who had not participated in the study, to judge the similarity of the results with their own experiences.

### Ethical considerations

Ethical approval of the research was received from the Ethics Committee of Isfahan University of Medical Sciences (ethical approval code: IR.MUI.Rec.1395.3.599). Informed consent, anonymity, confidentiality and the right of participants to leave the study at any time was preserved.

## Results

During data analysis, 150 codes and 20 sub-categories developed. The final five main categories were “help in maintaining the health of the mother and fetus”, “emotional support of mother”, “comprehensive participation of father in married life”, “preparing for safe delivery” and “postpartum support” (Table 2).

**Table 2:** Results of data analysis

Code	Sub-category	Category
• Providing settings for sufficient rest time for wife • Provide massages to wife during pregnancy	Physical care of wife during pregnancy	Help in maintaining the health of the mother and fetus
• Avoid smoking at home during pregnancy • Considering their wives’ condition during sexual intercourse	Maintaining the health of the mother and fetus	
• Reduce daily overtime and arrive home earlier • Continuous presence alongside spouse at end of pregnancy	Increase the presence of husbands at home during pregnancy	
• Make sure wife eats sufficient, nutritious and healthy food during pregnancy • Realize importance of feeding pregnant wife	Concern for the mother’s nutrition during pregnancy	
• Provide the necessary clothing and equipment for wife during pregnancy • Provide cost of perinatal care and paraclinical measures	Meeting the financial needs of the mother during pregnancy and delivery	
• Accompany mother to pregnancy classes • Accept an active role in health care decisions	Accompanying the mother to prenatal classes and during pregnancy check-ups, delivery and postpartum care	
• Be empathetic and companionable to wife in the event of complications in pregnancy • Understand the condition of wife in having nausea and vomiting during pregnancy • Be empathic and participate with wife in the event of a high risk pregnancy	Empathy with mother	Emotional support of mother
• Do not create tension at home during the perinatal period • Consider the emotional and physical conditions of the mother when inviting guests during pregnancy • Help the wife create a positive mental image of her body	Providing peace of mind for mother during pregnancy	
• Use proper language when talking to pregnant wife • Do not order wife about or be aggressive towards her during pregnancy	Practicing good behavior and manners during pregnancy	
• Verbally express appreciation of wife to tolerate pregnancy and childbirth • Show appreciation of wife by giving gifts during pregnancy and childbirth	Showing appreciation for his wife’s efforts during pregnancy and after delivery	
• Do not reduce attention to mother after childbirth (and increase attention to child) • Understand the emotional needs of wife in postpartum period	Provision of wife’s emotional needs after delivery	
• Correct management of postpartum blues after delivery • Provide mental relaxation for wife and child after delivery at home	Appropriate management of postpartum emotional problems	
• Participate in the care of the other children during pregnancy • Help with cooking and wash dishes	Husband’s participation in home and life affairs	Comprehensive participation of father in married life
• Reduce expectations regarding a wife’s duties about laundry and cooking • Avoid argument with wife about not being able to do housework or cook	Lower expectations about houswork of the wife during pregnancy	
• Accompany a working pregnant woman on the way to and from work • Prepare environment for wife to rest after working outside the home	Accompanying the working mother to and from work during pregnancy	
• Plan for a well-equipped childbirth center for delivery of baby • Transfer his wife to the hospital in-time	Planning for delivery	Preparing for safe delivery
• Active presence alongside wife during childbirth • Presence beside wife in the time after childbirth	Presence of the husband beside his wife during delivery	
• Be supportive of baby’s first breastfeeding in hospital • Provide kangaroo care if necessary after delivery	Husband’s participation in mother’s and infant’s care after delivery in the hospital	Postpartum support
• Provide companionship during breastfeeding at night • Provide a proper diet for a mother who is breastfeeding	Husband’s participation in breastfeeding	
• Take paternal leave to care for wife and baby in the early days after childbirth • Help by changing diapers and bathing baby	Husband’s participation in mother and infant care at home after delivery	

### Help in maintaining the health of the mother and fetus

The main goal of prenatal care is maternal and fetal health, and achieving this goal requires the father’s participation.This main category contained six sub-categories.

#### Physical care of wife during pregnancy

Most participants emphasized the physical care of the wife during pregnancy by the husband. They believed that husbands can support their wives by providing an appropriate setting and time in which the expectant mother can rest and relax, taking care of her at home and offering massages to reduce the tension of pregnancy. They also believed that husbands should not go to extremes in the care of their wives because this could have negative outcomes.

*“During pregnancy, husbands should physically support their wives because she is like a flower that requires round-the-clock protection so that it doesn’t wither. Physical assistance is also important. She should not handle heavy housework or be forced to perform heavy housework.” (Father, 39 years old)*

#### Maintaining the health of the mother and fetus

Female participants stated that husbands should avoid actions such as smoking that might harm the fetus and the mother. They also should consider their wives’ condition during sexual intercourse and cooperate with their wives to maintain a healthy lifestyle for a healthy pregnancy and outcome. The husbands did not talk about such issues.

*“One thing that men do not consider, especially during pregnancy, and which is common among the patients, is sex. If husbands could be made aware of the natural process of pregnancy and the changes that occur in the body of a pregnant woman, they would be able to overcome these issues easily.” (Midwife, 46 years old)*

#### Increase the presence of husbands at home during pregnancy

Most wives emphasized a need for their husbands’ presence in the home during pregnancy because of the mood changes that occur in the mother. They believed that this could help mothers to overcome some of the fears and concerns about pregnancy and give them peace of mind. By contrast, most husbands believed that working and earning income to cover the costs of perinatal care and paraclinical measures during pregnancy is a top priority for them.

#### Concern for the mother’s nutrition during pregnancy

Participants believed that the mother’s sufficient consumption of nutritious food with affect her health and the growth and development of the fetus and should be provided by the husband.

*“My husband is very concerned about my diet and what I eat. He says that now I am responsible for two lives and should be eating foods that are beneficial for me. He buys only nutritious foods and expects me to consume all of them.” (Pregnant woman, 30 years old)*

#### Meeting the financial needs of the mother during pregnancy and delivery

Most participants, especially pregnant housewives, were concerned about the economic role of men and considered it to be their most important role. They stated that the cost of the specific care and paraclinical measures required by pregnant women should be provided by their husbands. This will allow the mother to calmly focus on her own health and that of her baby without concern about the cost. In this case, husbands agreed with their wifes and felt that meeting the financial needs of the pregnancy was a priority.

*“From the economic point of view, it is evident that specific care, such as an ultrasound or blood tests, and the proper nutrition required by the mother are all costly. Because in our culture, the family budget is controlled by husbands, it should be provided by them.” (Pregnant woman, 30 years old)*

#### Accompanying the mother to prenatal classes and during pregnancy check-ups, delivery and postpartum care

Most female and some male participants emphasized the need for the fathers to accompany their wives to perinatal and delivery classes and considered this action and acceptance of their common role in making medical and care decisions as a male characteristic. Policymakers and healthcare professionals believed that improving the awareness of husbands to learn about the process of pregnancy and delivery could increase their concern about providing care for their wives and help prevent maternal and fetal complications.

### Emotional support of mother

The main need of pregnant women is husband’s emotional support. This main category contains six sub-categories.

#### Empathy with mother

Most participants felt that pregnancy is a stressful and challenging period for women and that father could improve this experience for their wives with their emotional support. Most female participants felt that a good husband is one who supports his wife under any condition. For example, a wife may not want sex or may avoid her husband because of vomiting and nausea or aversions caused by pregnancy or the pregnancy may high risk or there may be anomalies in the fetus. At such times, a husband should not deny emotional support for the mother or consider her to be at fault. By offering support, he takes this burden off his wife’s shoulders and helps her to tolerate the problems. Most expectant fathers were unaware of the changes caused by pregnancy and the need to empathize with their wives during this period because they considered pregnancy to be natural for women and not significantly different from non-pregnancy.

*“When there is a problem with a blood test or ultrasound, the wife should not have to cope with this problem all by herself; the husband should also participate.” (Midwife, 38 years old)*

#### Providing peace of mind for mother during pregnancy

All participants acknowledged that providing peace of mind for the wife during pregnancy is a primary need of mothers during the perinatal period. During pregnancy, men should show their wives that they are willing partners in the process of pregnancy. A husband should provide a safe and peaceful environment in the house, avoid tension and help his wife to have a positive image of her body. He should try to decrease stress and concerns felt by his wife by reassuring her, getting her to the hospital on time, providing the cost of delivery and participating in the care of the infant. He should also recognize the effects of her condition when visiting others or having guests and respects her decisions in this regard.

*“The concept of participation by the husband includes support and assurance that he realizes her problems and supports her. Although this is mostly emotional, she needs physical support less than she needs emotional support.” (Midwife, 28 years old)*

#### Practicing good behavior and manners during pregnancy

Respect for good behavior during pregnancy, the use of appropriate language, respect for the mother’s dignity, refraining from ordering her about and being aggressive towards her during pregnancy will preserve a woman’s self-esteem and empower her to be a mother.

*“Because women need support, aggression during pregnancy could cause severe damage.” (Policy maker, 36 years old)*

#### Showing appreciation for his wife’s efforts during pregnancy and after delivery

Most female and some male participants stated that husbands should appreciate their wives’ efforts during pregnancy and after delivery by buying her gifts or by verbally expressing appreciation. This woman has carried the fruit of their married life in her womb for 9 months and willingly tolerated the difficult conditions of pregnancy and delivery.

*“I am a municipal worker and am only at home for five hours a day. I could compensate for my lack of presence at home by apologizing and saying that I regret not being able to be with [my wife] more. For example, I don’t have a lot of money, but I took this flower from the park today for you.” (Father, 36 years old)*

#### Provision of wife’s emotional needs after delivery

The participants felt that husbands should provide more emotional support for their wives after delivery than during pregnancy so that the mother would not feel that all of the attention during pregnancy was actually for the child and not her.

*“Although the baby is a part of me, my priority should be the mother. I should focus more affection on her than on the child so that she doesn’t think, ‘he only wanted me for a child’.” (Father, 34 years old)*

#### Appropriate management of postpartum emotional problems

Female Participants believed that the postpartum period is important to the mother’s emotional health. Postpartum depression is a possibility that should be considered and managed by husbands to avoid damage to the emotional health of the mother and help her return to normal life.

*“The most important participation of men after delivery is emotional because postpartum depression might occur due to hormonal changes.” (Pregnant woman, 21 years old)*

### Comprehensive participation of father in married life

Husband’s participation in all matters from the beginning of married life, especially during pregnancy, was the desire of women and called for equal participation of father in care and home affairs. This category is contains three sub-categories.

#### Husband’s participation in home and life affairs

Most participants believed that during his wife’s pregnancy, the husband should participate in the cooking and cleanup and handle heavy household jobs. They should adopt a positive role in the care and rearing of the children and in preparing other children to accept the new infant.

“*During that period, I spent time with my wife for months. I took over responsibility for everything, even cooking, vacuuming and washing dishes. Sometimes when we had guests, I would ask one of them to come and help me cook the food.” (Father, 39 years old)*

#### Lower expectations about housework of the wife during pregnancy

Most female and some male participants believed that husbands should lower their expectations regarding their wives’ duties toward laundry and food preparation and should not argue with their wives if they fail to do the housework or cook. They should not have unrealistic expectations of their pregnant wives and should not force them to perform heavy jobs or cook when they are experiencing morning sickness (especially during the first trimester).

*“When he comes into the house and sees that I am not feeling well he should not expect me to prepare a great dinner for him. If one day I could not iron his clothes, it should not ignite an argument; he should understand my condition.” (Pregnant woman, 34 years old)*

#### Accompanying the working mother to and from work during pregnancy

Working pregnant women who participated in the study said that they would like their husbands to prepare a relaxing environment for them after working outside the house and accompany them to and from work. They believed that husbands should share equally in the housework and care of the children. When it is no longer possible for the wives to work, husbands should encourage their wives to take unpaid leave to avoid harm to them and their babies.

“*When a women is working, sometimes she would like her husband to tell her, ‘For the sake of your health and our child’s health, I don’t care if you work or not’. A woman might not leave her job when he says this, but it gives her strength of heart and feel that she could take unpaid leave whenever she wants because her health is more important.” (Policy maker, 36 years old)*

### Preparing for safe delivery

Planning for delivery and presence of the husband beside his wife during delivery are the features of this main category. This main category contains two sub-categories.

#### Planning for delivery

Most female and some male participants believed that, during pregnancy, the husband should plan with his wife for the place of delivery and choose a well-equipped childbirth center. He should be by his wife during the last days of pregnancy and take her to the hospital on time when necessary.

*“There is a room beside the labor room in which fathers can sit. When women understand that their husbands are nearby, they can relax better. This will decrease their labor pain and the child will be born in peace.” (Father, 45 years old)*

#### Presence of the husband beside his wife during delivery

Most female and some male participants stated their willingness for fathers to be present with their wives during labor and actively participate by providing massage to ease the pain and provide emotional support. Some participants emphasized the presence of the husband and possibly other children during the early hours after delivery to promote bonding between family members.

*“I believe that the husband should be by his wife’s side at all stages of delivery, during labor and delivery, and fulfill the needs of his wife, her nutrition, her emotional issues and even massage her.” (Midwife, 46 years old)*

### Postpartum support

Because of mother’s emotional changes in postpartum period, father’s support is much more needed. This main category contains three sub-categories.

#### Husband’s participation in mother’s and infant’s cares after delivery in the hospital

Participants emphasized the husband’s presence by his wife’s side immediately after delivery at the hospital. They believed that the father should be present during the primary care of the infant and, for a preterm neonate; he should provide emotional support for the mother and participate in kangaroo care. If the delivery has been complicated and the mother is not able to take of the infant, especially a preterm one, the father is the best replacement for performing kangaroo care.

*“The first night that my son was born, I stayed at the hospital with my wife and did everything by myself. My son slept for hours in my arms.” (Father, 33 years old)*

#### Husband’s participation in breastfeeding

Female Participants believed that husbands should provide support during breastfeeding so that mothers do not have to tolerate the difficulties of this process by themselves. As their wives breastfeed, especially at night, fathers can provide physical and emotional support for their wives to make this process enjoyable for her. When the mother is too tired, he should feed the infant using expressed breast milk. It is necessary for a husband to focus on proper nutrition for his breastfeeding wife to maintaining her health.

*“While I’m breastfeeding, he should get up and massage my back. So what if the father massages his wife’s back while she is breastfeeding?” (Midwife, 39 years old)*

#### Husband’s participation in mother and infant care at home after delivery

The participants stated that fathers should cooperate during the early days after delivery in the care of their wives and infants. There should be sensitivity to the possible occurrence of complications for the mother and the infant during this period. By his constant presence near the mother and infant and his care of them, a father will assure their safety and be able to prevent complications. If complications arise, the father must work with his wife to get treatment. The care of a newborn is not just the mother’s duty. The father should also care for, bathe and change diapers. If the infant requires phototherapy or other treatments due to preterm birth, he should accompany his wife.

*“I remember that when my child’s bilirubin increased, I went to the hospital at 2 am, borrowed a phototherapy device, brought it home and put my baby under it. I slept on one side of the device and my wife slept on the other side. When the baby was hungry or when it was time for medication, I would help her.” (Father, 39 years old)*

## Discussion

The present study was conducted to determine the concept of a husband’s participation in perinatal care from perspective of mothers, fathers, caregivers, managers and policymakers. Understanding the experiences and attitudes of the different participant to the issue of male participation in the perinatal period is an important step in understanding the changes that must be made to promote marital partnership. The results of the present study show that the most important aspects of male participation in perinatal care were to help maintain the health of the mother and fetus, provide emotional support for the wife during pregnancy, delivery and after delivery, to comprehensively participate in married life, preparing for a safe delivery and postpartum support.

The presence and participation of the husband is essential to completing a healthy pregnancy. The husband’s physical support, accompaniment and encouragement of a healthy lifestyle and in receiving perinatal care will improve the health of the mother and the neonate. Participants in a study by Simbar et al. also emphasized the need for fathers to participate in perinatal care, which is in line with results of the present study [20]. Davis et al. showed that the involvement of the fathers in reproductive health and maternal and child health services in countries with low and medium incomes leads to better health outcomes and has significant advantages for the health of mothers and infants health.

The emotional support of the husband was another aspect of participation in perinatal care that was proposed by the participants. A husband’s ability to empathize with his wife and provide a safe and peaceful environment free of aggression and restrictions was also discussed by women. The studies of Mortazavi, Mirzaii [21] and Simbar et al. [20] revealed that emotional support for women and her need for love and affection are the most important aspect of the father’s participation in perinatal care. A study in China showed that a husband’s emotional support was the most influential in decreasing the risk of postpartum depression [17]. This indicates that men and women have realized the importance of psychological health during the perinatal period and that the husband is the best provider of his wife’s emotional needs. It might the effect of information transmitted through social media [22].

The experiences of female participants in the present study emphasized the need to avoid blaming the wife for pregnancy complications or miscarriage. This is in part the result of the ignorance of men about male-related factors in the failure of pregnancy, insufficient knowledge about the process of pregnancy and delivery, their consideration of pregnancy and delivery as a feminine problem and, thus, a lack of a feeling of responsibility in this regard. Studies in Mexico and Kenya also revealed that when fathers placed the blame for a failed pregnancy on the mothers, it could hinder their recovery from psychological problems and have an adverse effect on the couple’s relationship [23].

All participating women and some of the men believed that the comprehensive participation of husbands in married life is necessary. They emphasized that the husband must participate in the housework, cooking and care of the children and in all decision-making during pregnancy, delivery and after delivery. This suggests an improvement in female awareness about their rights in married life and could also be the result of increased female employment and their contribution to the household economy, which causes them to believe that their husbands should participate in the housework. Changes through the generations and traditional beliefs about men along with a decrease in the role of patriarchy in society could explain the more positive attitude of men toward their participation in housework.

In line with the results of previous studies in Iran and Kenya, the present study found that most women emphasized that men should lower their expectations of their wives during pregnancy with regard to housework and cooking and that men emphasized their role in providing the necessities of life and making money [20, 21, 24]. It appears that the high cost of perinatal care and delivery and the generally low income of men, who may have multiple jobs, have made making money their priority role. In this regard, reducing the cost of delivery and providing public health insurance for pregnancy could relieve the concerns of men about providing for the high cost of pregnancy and delivery and increase their participation at home.

In addition, participants mentioned preparation for a safe delivery as another area in which the husband should participate. They stated that he, along with his wife, should plan for the place of delivery, accompany her at the time of delivery, transport her to the hospital on time and stay in the delivery room or, if not allowed, in the hospital at all times. Studies have shown that in most countries with high incomes, a great change has occurred regarding the presence of the father at the time of delivery. Reports have revealed that in England in 2010, 90% of fathers were present at their child’s birth [25].

Kaye et al. indicated that the definition of an ideal father from the perspective of the male participants was to provide support during labor [26]. Simbar et al. [20] showed that most participating women and half of the men listed accompanying the mother to the delivery room as a definition of the participation of a father. This suggests that cultural taboos about the non-involvement of men during delivery have faded, that women’s expectations of their husbands have increased and emphasizes the importance of the health of mothers and children.

Another aspect of participation of fathers that was emphasized was postpartum support. Studies have shown that the involvement of fathers in perinatal care and during the important and sensitive postpartum period facilitates the early onset of breastfeeding and exclusive breastfeeding [27, 28], increases the rate of immunization of the infant and develops a stronger father-child relationship later in the child’s life. It is also associated with positive cognitive, developmental and social behavior of the children [9, 29, 30], including improved weight gain in preterm infants and breastfeeding [31–34]. Modification of maternity and postpartum wards in hospitals to allow for the presence of fathers, changing the attitudes of health personnel and developing paternity leave plans for fathers can facilitate the participation of fathers and allow them to assume their responsibilities for the care of their wives and children.

Generalization of the findings in the present study, considering its qualitative approach, should be done cautiously. Although qualitative studies are not designed for the generalization of the results, they are useful for those who are willing to use the results while considering the limitations. By selecting participants with maximum differences, seeking the guidance and supervision of experts and the use of external reviews, the accuracy and transferability of the data can be increased.

## Conclusion

In the present study, the important aspects of men’s participation in perinatal care were found to include “help in maintaining the health of the mother and fetus”, “emotional support of mother”, “comprehensive participation of father in married life”, “preparing for safe delivery” and “postpartum support”. Approaches to the expansion of the role of husbands during the pregnancy, delivery and the postpartum period include education and development of cultural awareness. In addition, the participation of fathers during and after delivery in the hospital should be facilitated by creating appropriate spaces in the hospital and wards and educating healthcare personnel about the need for fathers to participate during pregnancy, delivery and after delivery. This requires the help of policymakers and managers of the health system.

## Additional files

Additional file 1: Interview guide during the face-to-face interviews with women who were pregnant or had just delivered for the study conducted on participation of fathers in perinatal care from the perspective of mothers, fathers, caregivers, managers and policymakers in Tabriz Town, Iran, 2017 (See methods section for further description). (DOCX 16 kb)

Additional file 2: Interview guide during the face-to-face interviews with husbands of pregnant women or husbands of women who had recently experienced delivery for the study conducted on participation of fathers in perinatal care from the perspective of mothers, fathers, caregivers, managers and policymakers in Tabriz Town, Iran, 2017 (See methods section for further description). (DOCX 16 kb)

Additional file 3: Interview guide during the face-to-face interviews with health care providers (midwives, gynecologists and nurses) for the study conducted on participation of fathers in perinatal care from the perspective of mothers, fathers, caregivers, managers and policymakers in Tabriz Town, Iran, 2017 (See methods section for further description). (DOCX 16 kb)

Additional file 4: Interview guide during the face-to-face interviews with deputy health managers and policymakers for the study conducted on participation of fathers in perinatal care from the perspective of mothers, fathers, caregivers, managers and policymakers in Tabriz Town, Iran, 2017 (See methods section for further description). (DOCX 16 kb)

Additional file 5: Interview guide during the focus group discussions (for pregnant women) for the study conducted on participation of fathers in perinatal care from the perspective of mothers, fathers, caregivers, managers and policymakers in Tabriz Town, Iran, 2017 (See methods section for further description). (DOCX 16 kb)

Additional file 6: Interview guide during the focus group discussions (for health care providers) for the study conducted on participation of fathers in perinatal care from the perspective of mothers, fathers, caregivers, managers and policymakers in Tabriz Town, Iran, 2017 (See methods section for further description). (DOCX 17 kb)

## Abbreviations

AIDS: Acquired Immune Deficiency Syndrome
ICPD: International Conference on Population and Development
STIs: Sexually Transmitted Infections
WHO: World Health Organization
