# Supplementary material for: Participation of father in perinatal care: a qualitative study from the perspective of mothers, fathers, caregivers, managers and policymakers in Iran
Source: BMC Pregnancy Childbirth. 2018 Jul 11;18:297. doi: 10.1186/s12884-018-1928-5 (PMC6042395; doi:10.1186/s12884-018-1928-5)
Supplement: Supplementary file 1 — Interview guide during the face-to-face interviews with women who were pregnant or had just delivered for the study conducted on participation of fathers in perinatal care from the perspective of mothers, fathers, caregivers, managers and policymakers in Tabriz Town, Iran, 2017 (See methods section for further description). (DOCX 16 kb) [file 12884_2018_1928_MOESM1_ESM.docx]

**Additional file 1:** Interview guide during the face-to-face interviews with women who were pregnant or had just delivered for the study conducted on participation of fathers in perinatal care from the perspective of mothers, fathers, caregivers, managers and policymakers in Tabriz Town, Iran, 2017 (See methods section for further description).

**Introduction:** *Aim, to create appropriate atmosphere*

- Name of the interviewer and affiliation
- Purpose of the study
- Consent to take part in the study
- Confidentiality, explain how the data will be used
- Interview will last approximately 30-60 minutes
- Audio recorded to ensure interviewer can fully engage in the interview

**Warm up questions:** *Aim\ make participants comfortable*

1. Please introduce yourself?
2. How old are you?
3. What is your education level?
4. What do you do?
5. How many pregnancies (childbearing) have you had before?
6. How many months pregnant are you? How long is it since your childbearing?
7. Can you please tell us your story of becoming a mother?

**Questions of an interview guide in individual interview with pregnant or childbearing women**

1. What is the meaning of the participation of fathers during pregnancy in your opinion?

2. Do/did you feel the need for your husband’s help during pregnancy?

3. How do you think your husband can help you during pregnancy?

4. What is the meaning of the participation of fathers during childbearing in your opinion?

5. Do you think it is necessary that the husband attends the delivery room during childbearing? Please explain.

6. What should have your husband done during your pregnancy and he hadn’t? (For women with a history of childbirth)

7. How do you think your husband can help you and your infant in the postpartum period?

8. Do you think the participation of your husband is essential in the postpartum period?

9. Who can help the mother in the postpartum period? And who are you expecting to get more help from in this regard?

10. Have you needed any kind of help in the postpartum period that your husband hadn’t met? What was it? (For women with a history of childbirth)
