# Supplementary material for: Participation of father in perinatal care: a qualitative study from the perspective of mothers, fathers, caregivers, managers and policymakers in Iran
Source: BMC Pregnancy Childbirth. 2018 Jul 11;18:297. doi: 10.1186/s12884-018-1928-5 (PMC6042395; doi:10.1186/s12884-018-1928-5)
Supplement: Supplementary file 2 — Interview guide during the face-to-face interviews with husbands of pregnant women or husbands of women who had recently experienced delivery for the study conducted on participation of fathers in perinatal care from the perspective of mothers, fathers, caregivers, managers and policymakers in Tabriz Town, Iran, 2017 (See methods section for further description). (DOCX 16 kb) [file 12884_2018_1928_MOESM2_ESM.docx]

**Additional file 2:** Interview guide during the face-to-face interviews with husbands of pregnant women or husbands of women who had recently experienced delivery for the study conducted on participation of fathers in perinatal care from the perspective of mothers, fathers, caregivers, managers and policymakers in Tabriz Town, Iran, 2017 (See methods section for further description).

**Introduction:** *Aim, to create appropriate atmosphere*

- Name of the interviewer and affiliation
- Purpose of the study
- Consent to take part in the study
- Confidentiality, explain how the data will be used
- Interview will last approximately 30-60 minutes
- Audio recorded to ensure interviewer can fully engage in the interview

**Warm up questions:** *Aim\ make participants comfortable*

1. Please introduce yourself?
2. How old are you?
3. What is your education level?
4. What do you do?
5. How many children do you have?
6. Is your wife pregnant or giving birth?
7. Can you please tell us your story of becoming a father?

**Questions of the interview guide in interviewing the fathers**

1. What do you think about the participation of fathers during pregnancy?
2. How do you think a man can help his wife during pregnancy, childbirth, and delivery?
3. What do you think in the participatory role of the father during childbirth and his presence in the delivery room? Please explain.
4. Do you give positive answers to all of the needs of your wife during pregnancy? Why?
5. Why do you think it is believed that pregnancy, childbearing and postpartum issues are women’s business?
6. Have you taken/ are you going to take a leave to care for your spouse? Please explain.
7. Have you or would you help your spouse in taking care of the baby?
8. What do you think about the role of the father in the postpartum period?
