# Supplementary material for: Participation of father in perinatal care: a qualitative study from the perspective of mothers, fathers, caregivers, managers and policymakers in Iran
Source: BMC Pregnancy Childbirth. 2018 Jul 11;18:297. doi: 10.1186/s12884-018-1928-5 (PMC6042395; doi:10.1186/s12884-018-1928-5)
Supplement: Supplementary file 4 — Interview guide during the face-to-face interviews with deputy health managers and policymakers for the study conducted on participation of fathers in perinatal care from the perspective of mothers, fathers, caregivers, managers and policymakers in Tabriz Town, Iran, 2017 (See methods section for further description). (DOCX 16 kb) [file 12884_2018_1928_MOESM4_ESM.docx]

# Additional file 4: Interview guide during the face-to-face interviews with deputy health managers and policymakers for the study conducted on participation of fathers in perinatal care from the perspective of mothers, fathers, caregivers, managers and policymakers in Tabriz Town, Iran, 2017 (See methods section for further description).

**Introduction:** *Aim, to create appropriate atmosphere*

- Name of the interviewer and affiliation
- Purpose of the study
- Consent to take part in the study
- Confidentiality, explain how the data will be used
- Interview will last approximately 30-60 minutes
- Audio recorded to ensure interviewer can fully engage in the interview

**Warm up questions:** *Aim\ make participants comfortable*

1. Please introduce yourself?
2. How old are you?
3. What is your education level?
4. What do you do?
5. How many children do you have?
6. Are you single or married?
7. What is your carrier experience?
8. What is your position?

**Questions of the interview guide in interviewing the deputy health** **managers and policy makers**

1. What is the meaning of the participation of fathers during pregnancy, childbearing and postpartum period in your opinion?
2. What do you think about the presence and participation of fathers in these kinds of caring activities?
3. As a manager or policy maker, which steps, in your opinion, are necessary for the enhancement of father’s participation in the pregnancy, childbirth or postpartum periods?
4. What measures have you taken to enhance the participation of fathers during pregnancy, childbirth or postpartum periods?
