# Supplementary material for: Participation of father in perinatal care: a qualitative study from the perspective of mothers, fathers, caregivers, managers and policymakers in Iran
Source: BMC Pregnancy Childbirth. 2018 Jul 11;18:297. doi: 10.1186/s12884-018-1928-5 (PMC6042395; doi:10.1186/s12884-018-1928-5)
Supplement: Supplementary file 5 — Interview guide during the focus group discussions (for pregnant women) for the study conducted on participation of fathers in perinatal care from the perspective of mothers, fathers, caregivers, managers and policymakers in Tabriz Town, Iran, 2017 (See methods section for further description). (DOCX 16 kb) [file 12884_2018_1928_MOESM5_ESM.docx]

**Additional file 5:** Interview guide during the focus group discussions (for pregnant women) for the study conducted on participation of fathers in perinatal care from the perspective of mothers, fathers, caregivers, managers and policymakers in Tabriz Town, Iran, 2017 (See methods section for further description).

**Introduction:** *Aim, to create appropriate atmosphere*

- Name of the interviewer and affiliation
- Purpose of the study
- Consent to take part in the study
- Confidentiality, explain how the data will be used
- Discussion will last approximately 60-90 minutes
- Audio recorded to ensure interviewer can fully engage in the interview

**Warm up questions:** *Aim\ make participants comfortable*

1. Please introduce yourself?
2. How old are you?
3. What is your education level?
4. What do you do?
5. How many children do you have?

**Questions of the interview guide in the focus group discussions of pregnant women**

1. What is your opinion about the participation of husbands during pregnancy? Please explain.
2. What is your opinion about the participation of husbands during childbearing?
3. What is your opinion about the participation of fathers in caring the mother and infant after childbearing?
4. In your opinion, how fathers can be involved in caring the mother and the infant after childbirth?
