# Supplementary material for: Participation of father in perinatal care: a qualitative study from the perspective of mothers, fathers, caregivers, managers and policymakers in Iran
Source: BMC Pregnancy Childbirth. 2018 Jul 11;18:297. doi: 10.1186/s12884-018-1928-5 (PMC6042395; doi:10.1186/s12884-018-1928-5)
Supplement: Supplementary file 6 — Interview guide during the focus group discussions (for health care providers) for the study conducted on participation of fathers in perinatal care from the perspective of mothers, fathers, caregivers, managers and policymakers in Tabriz Town, Iran, 2017 (See methods section for further description). (DOCX 17 kb) [file 12884_2018_1928_MOESM6_ESM.docx]

**Additional file 6:** Interview guide during the focus group discussions (for health care providers) for the study conducted on participation of fathers in perinatal care from the perspective of mothers, fathers, caregivers, managers and policymakers in Tabriz Town, Iran, 2017 (See methods section for further description).

**Introduction:** *Aim, to create appropriate atmosphere*

- Name of the interviewer and affiliation
- Purpose of the study
- Consent to take part in the study
- Confidentiality, explain how the data will be used
- Discussion will last approximately 60-90 minutes
- Audio recorded to ensure interviewer can fully engage in the interview

**Warm up questions:** *Aim\ make participants comfortable*

1. Please introduce yourself?
2. How old are you?
3. What is your education level?
4. What do you do?
5. How many children do you have?
6. Are you single or married?
7. What is your carrier experience?

**Questions of the interview guide in the focus group discussions of health care** **providers**

1. What is the meaning of the participation of fathers during pregnancy, childbearing and postpartum period in your opinion?
2. As a service provider, what do you think of the presence and participation of fathers in these kinds of caring activities?
3. As a service provider how can you help foster father’s participation in pregnancy, childbirth, and postpartum period?
4. What is the association of woman’s success in delivering or breastfeeding and the husband’ participation and help?
